# Manipulating the sleeping beauty mutase operon for the production of 1-propanol in engineered *Escherichia coli*

**DOI:** 10.1186/1754-6834-6-139

**Published:** 2013-09-28

**Authors:** Kajan Srirangan, Lamees Akawi, Xuejia Liu, Adam Westbrook, Eric JM Blondeel, Marc G Aucoin, Murray Moo-Young, C Perry Chou

**Affiliations:** 1 Department of Chemical Engineering, University of Waterloo, 200 University Avenue West, Waterloo, ON N2L 3G1, Canada

**Keywords:** Bifunctional aldehyde/alcohol dehydrogenases, Cyanocobalamin, Metabolic engineering, Methylmalonyl-CoA mutases, Propanol, Propionate, Sleeping beauty mutase operon

## Abstract

**Background:**

While most resources in biofuels were directed towards implementing bioethanol programs, 1-propanol has recently received attention as a promising alternative biofuel. Nevertheless, no microorganism has been identified as a natural 1-propanol producer. In this study, we manipulated a novel metabolic pathway for the synthesis of 1-propanol in the genetically tractable bacterium *Escherichia coli*.

**Results:**

*E. coli* strains capable of producing heterologous 1-propanol were engineered by extending the dissimilation of succinate via propionyl-CoA. This was accomplished by expressing a selection of key genes, i.e. (1) three native genes in the sleeping beauty mutase (Sbm) operon, i.e. *sbm*-*ygfD*-*ygfG* from *E. coli*, (2) the genes encoding bifunctional aldehyde/alcohol dehydrogenases (ADHs) from several microbial sources, and (3) the *sucCD* gene encoding succinyl-CoA synthetase from *E. coli*. Using the developed whole-cell biocatalyst under anaerobic conditions, production titers up to 150 mg/L of 1-propanol were obtained. In addition, several genetic and chemical effects on the production of 1-propanol were investigated, indicating that certain host-gene deletions could abolish 1-propanol production as well as that the expression of a putative protein kinase (encoded by *ygfD/argK*) was crucial for 1-propanol biosynthesis.

**Conclusions:**

The study has provided a novel route for 1-propanol production in *E. coli*, which is subjected to further improvement by identifying limiting conversion steps, shifting major carbon flux to the productive pathway, and optimizing gene expression and culture conditions.

## Background

The majority of the world’s energy requirements are currently met through unfettered use of carbonaceous fossil fuels. However, mounting environmental and socioeconomic concerns associated with exploiting these resources have led to the exploration of more sustainable and environmentally friendly energy forms, in particular biofuels [1]. While ethanol, one of the most common and successful biofuels today, almost possesses established economic niches within energy markets, significant attention is being directed towards the production of longer-chain alcohols, such as 1-butanol and 1-propanol [2,3]. These longer-chain alcohols tend to have a higher energy content, lower hygroscopicity, and water solubility; and are compatible with existing transportation infrastructures and pipelines [4].

In addition to being a potential biofuel, 1-propanol serves as an important solvent and chemical for relevant industrial applications [5]. Up to now, the production of 1-propanol primarily relies on chemical synthesis and no microbial cells have been identified as a natural 1-propanol producer. Nevertheless, recent advances in synthetic biology and metabolic engineering have enabled biological production of 1-propanol using various non-natural but genetically tractable microorganisms, among which *Escherichia coli* is the most common. It is critical to identify potential synthetic pathways and enzymes relevant to the target metabolite (i.e. 1-propanol) heterologously produced in a non-native microbial host. For example, Atsumi et al., [2] devised a synthetic approach to convert 2-ketobutyrate to produce 1-propanol in a genetically engineered *E. coli* strain through a non-fermentative biosynthetic pathway mediated by a promiscuous 2-ketoacid decarboxylase and an aldehyde/alcohol dehydrogenase (ADH). The conversion bioprocess was further enhanced using an evolved citramalate pathway [6]. On the other hand, Choi et al., [7] demonstrated the production of 1-propanol by grafting a pathway containing several key genes for further conversion of l-threonine into 1-propanol in an engineered l-threonine overproducing *E. coli* strain. Jain and Yan [5] reported the production of 1-propanol in *E. coli* by expanding the 1,2-propanediol pathway with two steps mediated by a novel 1,2-propanediol dehydratase and an ADH. More recently, Shen and Liao [8] combined the native threonine pathway and a heterologous citramalate pathway for synergistic production of 1-propanol in *E. coli*. In addition to the aforementioned *E. coli* platforms, Deng and Fong [9] explored direct conversion of untreated plant biomass to 1-propanol using an engineered *Thermobifida fusca* strain.

Herein, we present an alternative novel biosynthesis of 1-propanol by manipulating the sleeping beauty mutase (Sbm) operon in *E. coli*. This four-gene operon (*sbm*-*ygfD*-*ygfG*-*ygfH*) encodes various enzymes involved in a cobalamin-dependent metabolic pathway for decarboxylation of succinate into propionate [10]. The metabolic context of the Sbm-pathway remains ambiguous, but is suspected to be involved in the assimilation of unusual carbon sources, such as succinate and propionate. Moreover, eponymous to its name, the operon genes are hardly expressed possibly due to an inactive or weak promoter-operator system [11,12]. Three of the encoded proteins from this operon are identified to be members of the crotonase superfamily, namely (1) *sbm* encoding a cobalamin-dependent methylmalonyl-CoA mutase (or Sbm; **s**leeping **b**eauty **m**utase), which catalyzes the isomerization of succinyl-CoA to l-methylmalonyl-CoA; (2) *ygfG* encoding a methylmalonyl-CoA decarboxylase (YgfG), which catalyzes the decarboxylation of methylmalonyl-CoA to propionyl-CoA; and (3) *ygfH* encoding a propionyl-CoA::succinate transferase (YgfH) [13]. The *ygfD* gene encodes a putative protein kinase (YgfD/ArgK) whose function remains unclear. However, YgfD could potentially interact with Sbm to form a multi-subunit complex [14]. Although the structure, function, and relationship of these enzymes have been characterized, hardly any work has been performed for their practical application.

**Figure 1:**
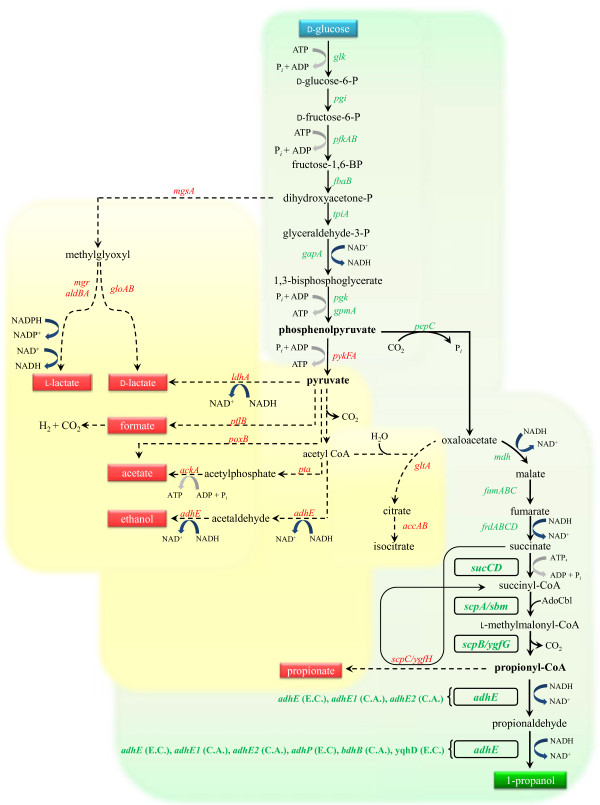
**The genetically engineered central metabolic pathway under anaerobic conditions showing the activation of the Sbm operon *(sbm***, ***ygfD***, ***and ygfG)*, and the expression of various *adhEs *used in this study.** Red colored gene names above or beside dashed lines represent diverting pathways; metabolites in red boxes are unwanted. Genes in green represent the necessary genes for 1-propanol conversion from glucose; those that are in bold font and boxed represent genes expressed via episomal plasmids.

**Figure 2:**
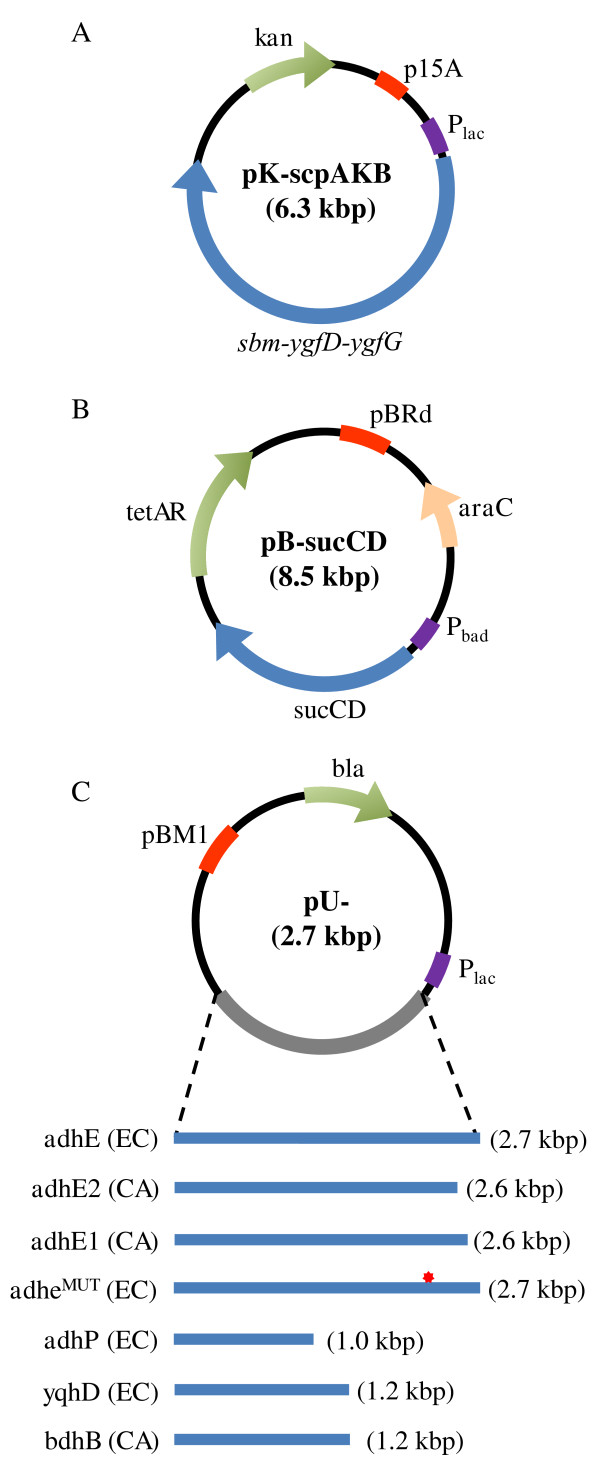
**A schematic representation of the triple-plasmid expression systems utilized for 1-propanol production.** All strains have **(A)** *sbm-ygfD-ygfG* cloned into pK184 under the control of p_*lac*_ as well as, **(B)** *sucCD* cloned into pBBR1MCS-3 under the control of the arabinose inducible p_*araB*_. In addition to these, each strain has **(C)** pUC19 containing one of the seven listed alcohol dehydrogenases. The red star in the adhE^MUT^(EC) represents the E (glu)→K (lys) mutation at amino acid residue 568.

In this study, we demonstrated the production of 1-propanol using engineered *E. coli* strains with an activated Sbm operon for extended dissimilation of succinate (see Figure 1 for relevant pathways). First, three *E. coli* genes of *sbm*, *ygfD*, and *ygfG* were assembled as a single operon and then were expressed to convert succinyl-CoA to propionyl-CoA. Second, the genes encoding bifunctional ADHs from various microorganisms were cloned and expressed to convert propionyl-CoA to 1-propanol. We further channeled carbon flux towards the 1-propanol-producing pathway by expressing *sucCD* (encoding succinyl-CoA synthetase) from *E. coli*. These biosynthetic strategies were implemented into *E. coli* based on the construction of triple-plasmid expression systems (Figure 2) to facilitate the evaluation of suitable pathways. The 1-propanol-producing capacity of these metabolically engineered *E. coli* strains were evaluated under anaerobic cultivation conditions. The exometabolome of the culture was analyzed using 1-dimensional hydrogen nuclear magnetic resonance (1D-^1^H-NMR) spectroscopy with more than thirty metabolites being identified. In addition, we investigated several genetic and chemical effects associated with 1-propanol production in engineered *E. coli*.

## Results

### Construction of propanogenic *E. coli* strains for 1-propanol production

Based on the proposed novel pathway for the production of 1-propanol (Figure 1), the intracellular pool of propionyl-CoA, a rare metabolite in *E. coli*, should be first increased to promote its subsequent conversion to 1-propanol. To do this, genes encoding methylmalonyl-mutase (Sbm), arginine kinase (YgfD/ArgK), and methylmalonyl-CoA decarboxylase (YgfG) from the Sbm operon were cloned and expressed under the control of the P_*lac*_ promoter from plasmid pK-scpAKB. To convert the increased pool of propionyl-CoA to 1-propanol, the gene encoding a common bifunctional ADH from *C. acetobutylicum* was cloned and expressed under the control of the P_*lac*_ promoter from plasmid pU-adhE2(CA). While the wild-type strain of BW25141 showed no sign of propionate or 1-propanol production, approximately 47 mg/L of 1-propanol was detected for WT-adhE2(CA)^2^ when glucose was used as the sole carbon source (Table 1), implying that the implemented 1-propanol production pathway was functioning. A potential factor limiting the overall production of 1-propanol was perceived to be the abundance of various precursors, such as succinate and succinyl-CoA. To investigate this, the gene encoding *E. coli* succinyl-CoA synthetase (*sucCD*) gene was cloned and expressed under the control of the P_*araB*_ promoter from plasmid pB-sucCD. Compared to WT-adhE2(CA)^2^, a significant increase in both propionate and 1-propanol production was observed for WT-adhE2(CA)^3^ (Table 1), implying that the conversion catalyzed by succinyl-CoA synthetase can limit the production of 1-propanol under shake flask culture conditions . On the other hand, the production of propionate and 1-propanol was further extended for WT-adhE2(CA)^3^ when 4 g/L succinate was supplemented in the cultivation medium (Table 1), implying that succinate could also be a key precursor limiting 1-propanol production.

**Table 1:** **1-Propanol and other metabolite titers (mg/L) in reduced M9 minimal media using *E. coli *strain BW25141 transformed with appropriate plasmids**

**Strain**	**Carbon source**	**Metabolite titers (mg/L)**					
		**Succinate**	**Lactate**	**Acetate**	**Propionate**	**Ethanol**	**1-Propanol**
**Control**							
BW25141	Glucose	307 ± 36	128 ± 11	4436 ± 250	—	3021 ± 156	—
**Experimental**							
WT-adhE2(CA)^2^	Glucose	264 ± 8	2601 ± 642	2961 ± 72	Trace	2640 ± 170	47 ± 2
WT-adhE2(CA)^3^	Glucose	231 ± 11	1877 ± 303	2653 ± 55	51 ± 14	3199 ± 283	103 ± 16
WT-adhE2(CA)^3^	Glucose and succinate	2200 ± 172	2293 ± 2970	3699 ± 352	123 ± 21	2774 ± 297	168 ± 39
WT-adhE2(CA)^3^-∆ygfD	Glucose	269 ± 94	3970 ± 1367	2527 ± 142	52 ± 8	1999 ± 104	37 ± 1

Cultures were induced at an O.D_600_ of 15. Strains were cultivated anaerobically at 37°C for 72 h. Carbon sources: 20 g/L glucose and 4 g/L succinate where indicated. All experiments were performed in triplicate.

To further characterize this pathway, we investigated the dispensability of YgfD/ArgK, a gene product from the Sbm operon, for 1-propanol production. To do this, we excised the YgfD/ArgK coding region from plasmid pK-scpAKB. The resulting plasmid pK-scpAB was used to replace pK-scpAKB in WT-adhE2(CA)^3^ to form WT-adhE2(CA)^3^-∆ygfD. While 1-propanol production was detected in the WT-adhE2(CA)^3^-∆ygfD culture, the titer was approximately one third that of WT-adhE2(CA)^3^ (Table 1). Interestingly, the propionate concentrations from the two strains of WT-adhE2(CA)^3^ and WT-adhE2(CA)^3^-∆ygfD were approximately the same. From these results, we assume that the presence of YgfD/ArgK can be crucial for 1-propanol production.

### Manipulation of cultivation conditions

**Figure 3:**
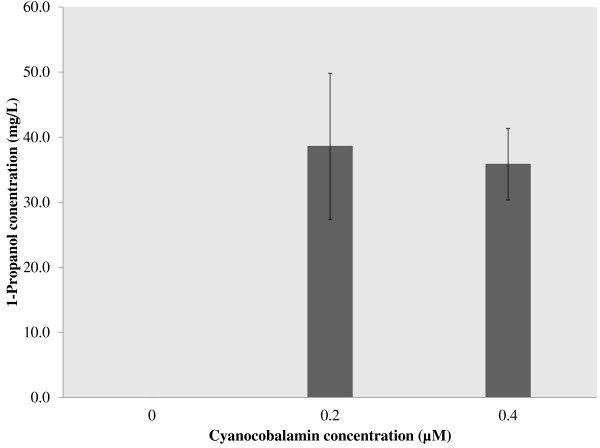
**The effect of cyanocobalamin concentration on 1-propanol production in strain WT-adhE2(CA)**^**3**^**.** 1-Propanol production is dependent on the exogenous supplementation of cyanocobalamin and saturation occurs at concentrations above 0.2 μM. Strains were cultivated anaerobically in reduced M9 minimal media with 20 g/L of glucose at 37°C for 72 h. All experiments were performed in triplicate.

As mentioned above, the production of 1-propanol can be limited by the structural rearrangement of succinyl-CoA into l-methylmalonyl-CoA. The catalytic activity of the enzyme responsible for this conversion, Sbm, is dependent on the availability of cyanocobalamin [15]. While *E. coli* encodes several cobalamin-dependent mutases and possesses receptors specifically for uptake of vitamin B_12_ (which is the active form of cyanocobalamin) [16], the organism neither produce cyanocobalamin *in vivo* nor require it for cell growth [17]. Using WT-adhE2(CA)^3^ as the host/vector system, it was observed that 1-propanol can be produced only when a threshold concentration of cyanocobalamin of 0.2 μM was supplemented in the cultivation medium. Using several cyanocobalamin concentrations less than 0.2 μM either significantly reduced or even abolished 1-production (Figure 3). As a result, this cyanocobalamin concentration of 0.2 μM was used for all cultivations.

**Figure 4:**
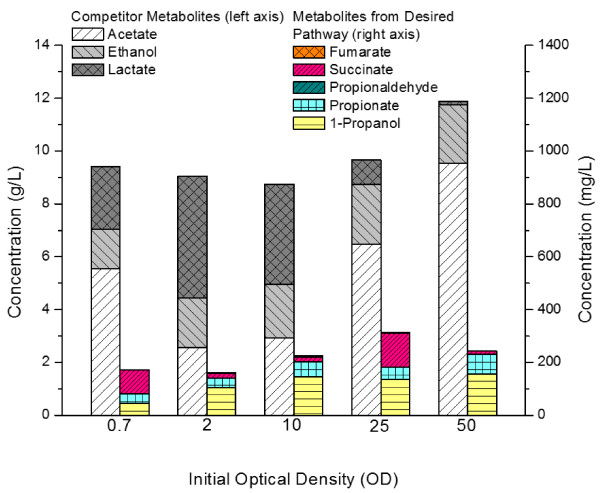
**End point secretion profile of major end products from anaerobic fermentations of WT-adhE2(CA)**^**3 **^**at five optical densities (OD600), profiled by 1D-**^**1**^**H-NMR.** Major end products that are competitor metabolites to the production of 1-propanol are quantified by the left axis. Products detected along the desired metabolic pathway towards formation of 1-propanol are quantified by the right axis. Strain was cultivated anaerobically in reduced M9 minimal media with 20 g/L of glucose at 37°C for 72 h.

**Figure 5:**
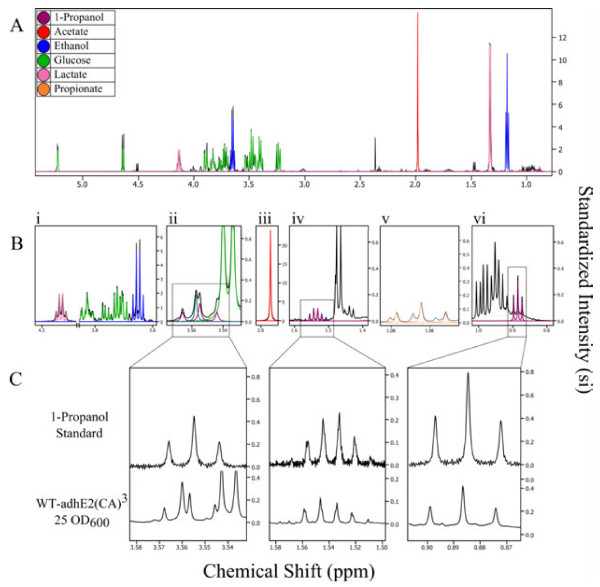
**Single dimension hydrogen NMR spectra scanned at 600 MHz from samples of *E. coli *supernatant from strain WT-adhE2(CA)**^**3**^**.** Strain was cultivated anaerobically in reduced M9 minimal media with 20 g/L of glucose at 37°C for 72 h. Culture samples were then centrifuged for 3 min at 13,000 × g to recover the supernatant fraction for analysis. **A)** The 25 OD_600_ spectrum profiled for metabolites using Chenomx Suite 7.5. **B)** Zoomed in panels from part A, identifying the three peak clusters of 1-propanol and major end-product metabolites. From left to right the panels show: i. lactate, glucose and ethanol peaks, ii. convolution of glycine spectra with that of the first 1-propanol peak cluster, iii. acetate, iv. the unobscured second peak cluster of 1-propanol, v. propionate, vi. the third peak cluster of 1-propanol. **C)** Zoomed in panels from part B of the three 1-propanol peak clusters from pure solution standard and supernatant of WT-adhE2(CA)^3^ grown at 25 OD_600_.

Studies were conducted to investigate the effects of various operating parameters on cultivation performance, particularly 1-propanol titer. WT-adhE2(CA)^3^ was grown aerobically and then resuspended in five different optical cell densities for anaerobic fermentation and 1-propanol production. Typical major fermentation metabolites, including ethanol, lactate, and acetate, as well as those relevant to the proposed pathway, including succinate, 1-propanol, and propionate, were detected in extracellular medium samples and their titer distributions under various culture conditions are summarized in Figure 4. While the distribution of two major metabolites of acetate and lactate appears to be affected by suspension cell density, the sum of their titers remained rather constant at approximately 8 g/L. Such high levels of major metabolites can potentially inhibit cell growth during anaerobic fermentation. Interestingly, the titer of the other major metabolite ethanol was minimally affected by suspension cell density by maintaining at approximately 2 g/L. Metabolites associated with the 1-propanol-producing pathway were considered minor and their titer distribution was also affected by suspension cell density. 1-Propanol titer reached a peak level at approximately 150 mg/L when suspension cell density was higher than 10 OD_600_. Considering the above effects, suspension cell density at 25 OD_600_ was chosen for all characterization experiments in this study. In addition to HPLC analysis, metabolites of interest were also analyzed by NMR, either qualitatively or quantitatively, based on their unique spectral signature and the results of a representative culture sample are summarized in Figure 5. In particular, the spectral signature associated with 1-propanol, i.e. the three peak clusters, was mapped to verify the production of 1-propanol (Figure 5C).

### Effects of various ADHs on 1-propanol production

Based on reported biosynthetic pathways of several alcohols (particularly long-chain alcohols) [18,19], the sequential reduction of propionyl-CoA to propionaldehyde and then to 1-propanol via a bifunctional ADH can represent a key step limiting the overall production of 1-propanol. In addition to *C. acetobutylicum* bifunctional ADH (AdhE2), various other ADHs were investigated in this study. *E. coli* has several ADHs, including AdhE, AdhP, YqhD, EutG, and YiaY [18]. To evaluate the effects of these endogenous ADHs on 1-propanol production, an *E. coli* strain of WT2, similar to WT-adhE2(CA)^3^ but without episomal expression of AdhE2, was derived. This strain, though harboring its native ADHs, failed to produce any detectable amount of 1-propanol after 72 h of cultivation (data not shown). The results suggest that 1-propanol production was primarily mediated by *C. acetobutylicum* AdhE2 in WT-adhE2(CA)^3^. In principle, 1-propanol should be detected in WT2, since bioinformatics databases such as BRENDA [20] report that certain *E. coli* ADHs also possess affinity for either propionyl-CoA and/or propionaldehyde as potential substrates. The abolishment of 1-propanol production in WT2 may be attributed to the very low basal levels of the native ADHs present in the cell with higher affinities for other substrates.

To further study the effects of various *E. coli* ADHs on 1-propanol production, we respectively cloned the *adhE*, *adhP*, and *yqhD* genes for episomal expression (Figure 2) and the results are summarized in Table 2. Amongst the native ADHs, YqhD and AdhP were of particular interest because of their affinity for medium-to-long chain substrates [18]. Titers of 1-propanol detected in the WT-adhP(EC) and WT-yqhD(EC) cultures were ~25% less than that in WT-adhE2(CA)^3^. Note that both YqhD and AdhP are unifunctional ADHs and thus lack an acetaldehyde dehydrogenase domain at the carboxyl end. The bifunctional AdhE of *E. coli* (encoded by the *adhE* gene) was also evaluated. However, the plasmid containing the *E. coli adhE* gene cannot be transformed into *E. coli* cells since episomal expression of the endogenous AdhE appears to be physiologically toxic. To circumvent this limitation, we derived an aerotolerant mutant of AdhE, which was previously documented to be less toxic to *E. coli* cells [21], and the corresponding propanogenic strain, i.e. WT-AdhE^MUT^(EC), could produce 1-propanol, but only at a level similar to WT-adhP(EC) and WT-yqhD(EC) (Table 2). These results suggest that the activities of *E. coli* ADHs towards propionyl-CoA or propionaldehyde are less than *C. acetobutylicum* AdhE2 under similar cultivation conditions. Moreover, the utilization of a unifunctional or bifunctional ADH seems to have no major effects on 1-propanol biosynthesis. In addition to AdhE2, two alternative ADHs from *C. acetobutylicum*, i.e. AdhE1 and BdhB, which are involved in butanol production during Clostridia solventogenesis phase [22], were examined for their effects on 1-propanol production using WT-adhE1(CA) and WT-bdhB(CA) (Table 2). The 1-propanol titer of the WT-adhE1(CA) culture was ~25% lower than that of WT-adhE2(CA)^3^, whereas WT-bdhB(CA) demonstrated a 1-propanol capacity similar to WT-adhE2(CA)^3^ (Table 2). The results suggest that 1-propanol biosynthesis in *E. coli* can be mediated by a variety of ADHs and the intracellular levels of these ADHs appear to be critical to drive 1-propanol production under shake flask culture conditions.

**Table 2:** **Comparison of 1-propanol production titers and other metabolites (mg/L) by expression of several ADHs in *E. coli *strain BW25141, transformed with appropriate plasmids**

**Strain**	**Metabolite titers (mg/L)**					
	**Succinate**	**Lactate**	**Acetate**	**Propionate**	**Ethanol**	**1-Propanol**
**Control**						
BW25141	307 ± 36	128 ± 11	4436 ± 250	—	3021 ± 156	—
**Experimental**						
WT-adhE2(CA)^3^	231 ± 11	1877 ± 303	2653 ± 55	51 ± 14	2774 ± 297	103 ± 16
WT-adhP(EC)	239 ± 57	2986 ± 498	2545 ± 89	100 ± 18	3192 ± 80	84 ± 7
WT-yqhD(EC)	Trace	3322 ± 920	3818 ± 826	29 ± 67	3469 ± 538	69 ± 10
WT-adhE^MUT^(EC)	Trace	3762 ± 393	2164 ± 64	Trace	4016 ± 83	74 ± 6
WT-adhE1(CA)	Trace	411 ± 120	4247 ± 198	71 ± 10	4397 ± 403	76 ± 11
WT-bdhB(CA)	150 ± 131	2139 ± 474	2329 ± 21	67 ± 22	3455 ± 169	109 ± 6

Cultures were suspended in reduced M9 minimal media and induced at an O.D_600_ of 15. Strains were cultivated anaerobically at 37°C for 72 h. Glucose (20 g/L) was used as the sole carbon source and all experiments were performed in triplicate.

### Effects of host-gene deletions on 1-propanol production

While 1-propanol production based on this novel pathway in *E. coli* is feasible, the titer and yield can be potentially limited by the accumulation of major metabolites of lactate, acetate, and ethanol (Figure 4). Hence, we also explored deletion of several host genes involved in the production of these metabolites, specifically *adhE* encoding AdhE, *pta* encoding phosphotransacetylase, and *ldhA* encoding lactate dehydrogenase, and the results are summarized in Table 3. Deletion of *adhE* (in WT-∆*adhE*) reduced the production of ethanol significantly compared to wild-type BW25141. However, the 1-propanol-producing capacity of WT-∆*adhE* appears to be completely abolished, even after being transformed with the triple-plasmid expression system for activation of the Sbm pathway (data not shown). On the other hand, deleting *pta* (in WT-∆*pta*) resulted in marked growth retardation though the acetate levels were significantly reduced, compared to wild-type BW25141, with the main fermentative byproduct being lactate. Similar to WT-∆*adhE*, WT-∆*pta* was also incapable of producing 1-propanol when being transformed with the triple-plasmid expression system (data not shown). Deletion of *ldhA* (in WT-∆*ldhA*) significantly reduced lactate titers, with superior cell growth compared to wild-type BW25141 under aerobic conditions. In contrast to the previous two mutant strains, WT-∆*ldhA* retained the 1-propanol-producing capacity upon its transformation with the triple-plasmid expression system. Nevertheless, the 1-propanol titers for these expression systems were approximately half of that for WT-adhE2(CA)^3^ (Table 3). Note that both ethanol and acetate titers for these WT-∆*ldhA* expression systems were significantly higher than WT-adhE2(CA)^3^, implying that the carbon flux was not properly channeled into the 1-propanol-producing pathway. Furthermore, while WT-∆*ldhA* expression systems were competent producers of 1-propanol, certain double (i.e. ∆*ldhA* ∆*adhE*) and triple mutant (i.e. ∆*ldhA* ∆*adhE* ∆*pta*) counterparts failed to produce the target metabolite under shake flask culture conditions (data not shown).

**Table 3:** Secretion profile of the metabolites produced (mg/L) by various knock out strains with or without appropriate plasmids

**Strain**	**Metabolite titers (mg/L)**					
	**Succinate**	**Lactate**	**Acetate**	**Propionate**	**Ethanol**	**1-Propanol**
**Controls**						
BW25141	307 ± 36	128 ± 11	4436 ± 250	—	3021 ± 156	—
WT-∆*adhE*	Trace	99 ± 17	4646 ± 705.2	—	1936 ± 741.9	—
WT-∆*pta*	776 ± 57	7259 ± 14	694 ± 196	—	3927 ± 691	—
WT-∆*ldhA*	187 ± 7	195 ± 14	3960 ± 151	—	6128 ± 80	—
**Experimental**						
WT-adhE2(CA)^3^	231 ± 11	1877 ± 303	2653 ± 55	51 ± 14	2774 ± 297	103 ± 16
∆*ldhA*- adhE(EC)	206 ± 49	63 ± 3	4181 ± 550	—	6209 ± 183	42 ± 4
∆*ldhA*- adhE2(CA)	247 ± 64	77 ± 4	4210 ± 292	—	6713 ± 270	57 ± 1
∆*ldhA*- adhE1(CA)	256 ± 106	81 ± 10	3696 ± 652	—	5863 ± 9	45 ± 10
∆*ldhA*- adhE^MUT^(EC)	243 ± 8	79 ± 7	3814 ± 26	—	6021 ± 104	60 ± 9
∆*ldhA*- adhP(EC)	208 ± 115	190 ± 16	4488 ± 126	—	6124 ± 119	65 ± 2
∆*ldhA*-yqhD(EC)	145 ± 49	99 ± 16	4145 ± 14	—	5732 ± 77	38 ± 1
∆*ldhA*- bdhB(CA)	212 ± 50	89 ± 12	4351 ± 204	—	5652 ± 195	41 ± 4

Cultures were suspended in reduced M9 minimal media and induced at an O.D600 of 15. Strains were cultivated anaerobically at 37°C for 72 h. Glucose (20 g/L) was used as the sole carbon source and all experiments were performed in triplicate.

## Discussion

To date, metabolic engineering of *E. coli* for 1-propanol biosynthesis has been conducted through two major pathways, i.e. (1) the keto-acid biosynthetic pathway [6-8] and (2) the extended 1,2-propanediol pathway [5]. Unlike these approaches, our strategy focused on activation of the endogenous but often silent Sbm operon for extended conversion of succinate into 1-propanol. The 1-propanol-producing capacity was implemented by transforming a wild-type *E. coli* strain, BW25141, with three plasmids respectively harboring the Sbm operon genes (with the exception of *ygfG*), *sucCD*, and *adhE2* for expression of these key genes. Using the metabolically engineered strains for anaerobic fermentation, we obtained 1-propanol titers up to 150 mg/L which is comparable to those of other studies [5,9]. In addition, we identified several potential factors limiting 1-propanol production, in particular the abundance of precursors and the conversion step catalyzed by a bi-functional alcohol/aldehyde dehydrogenase. While it is possible to perform this biotransformation aerobically, anaerobic cultivation was chosen for two reasons. Firstly, the two TCA intermediates of succinate and succinyl-CoA are the precursors for 1-propanol biosynthesis and their abundance can potentially limit 1-propanol production. Under anaerobic, but not aerobic, conditions, *E. coli* generates both succinate and succinyl-CoA as fermentation end products via a reductive reverse TCA pathway (Figure 1). Secondly, potential oxygen-sensitivity of AdhE2 and other ADHs is another limitation for oxygenic production of 1-propanol.

While the expression of enzymes encoded by the Sbm operon is potentially detectable, their levels are far too low to form a functional pathway [13,14,23]. Moreover, due to *E. coli*’s inability to produce coenzyme B_12_, the expressed Sbm remains as an inactive apo-enzyme, but nano-molar supplementation of cyanocobalamin can result in the formation of active Sbm [24,25]. Our observations of no detectable titers of propionate and 1-propanol for wild-type BW25141 as well as the production of 1-propanol upon heterologous expression of the Sbm operon genes with proper supplementation of cyanocobalamin was associated with the activation of the Sbm-pathway. While the activated Sbm-pathway can result in 1-propanol production, the expression of SucCD was deemed crucial to increase the succinyl-CoA pool and consequently the 1-propanol titer. In addition, 1-propanol production was enhanced by exogenous supplementation of succinate. These results suggest that 1-propanol production can be limited by the availability of various precursors and key enzymes along this 1-propanol-producing pathway.

While the metabolic context for the three enzymes encoded by the four-gene Sbm operon, i.e. Sbm, YgfG, and YgfH, has been unraveled, the biological role of the other member, i.e. YgfD/ArgK, remains ambiguous. Earlier studies determined that YgfD/ArgK is a putative arginine kinase interacting with Sbm *in vivo* and *in vitro*[14] and involved in the phosphorylation of periplasmic binding proteins for amino acid translocation [11]. The activity of YgfD/ArgK was shown to be potentially essential for 1-propanol biosynthesis since the 1-propanol titer was significantly reduced by the *ygfD/argK* deletion. Interestingly, propionate production was hardly affected by the *ygfD/argK* deletion, and this result is consistent with a previous report [26], where propionate was derived from fatty acids by expressing the Sbm-operon genes excluding *ygfD/argK* in an engineered *E. coli* strain.

A selection of native and non-native ADHs were heterologously expressed for evaluation of their effects on 1-propanol-producing capacity of various metabolically engineered *E. coli* strains, with AdhE2 and BdhB being identified as the most prominent ones for 1-propanol production. Nevertheless, our consistent observation that ethanol titers were significantly higher than 1-propanol implies that propionyl-CoA or propionaldehyde might have less affinity towards ADHs than acetyl-CoA or acetaldehyde. Several native *E. coli* ADHs (e.g. YqhD, AdhP, and AdhE^MUT^) were also active in driving 1-propanol production, but in a much lower titer. In particular, the generation of the aerotolerent AdhE mutant (AdhE^MUT^) opens an avenue for aerobic production of 1-propanol. Under anaerobic conditions, the maximum theoretical yield (on the molar basis) of 1-propanol from glucose is less than one due to limited NADH availability. Thus, developing an oxygenic production system would be beneficial as it increases the carbon throughout whilst improving cell growth and physiology.

Under anoxic conditions for anaerobic fermentation in *E. coli*, the carbon flux at the PEP node favors reduction into pyruvate rather than carboxylation into oxaloacetate (OAA), with lactate, acetate, and ethanol as major metabolites (Figure 1). Note that there are four NADH-consuming steps along the 1-propanol-producing pathway downstream of phosphoenolpyruvate (PEP), whereas only one or two NADH-consuming steps for the other pathways associated with the major metabolites. The anaplerotic reactions within the metabolic network are optimized in order to balance the cell’s energy budget and electrons. Consequently, only ~10% of glucose consumed is channeled towards succinate and cell mass [27]. Our results suggest that the production of 1-propanol was potentially hampered by the inherent limitation in succinate production and a metabolic deficiency in NADH generation. Interestingly, propionate was also concomitantly produced with 1-propanol in our metabolically engineered strains (Tables 1 and 2). Additional studies are needed to elucidate the dichotomy between 1-propanol and propionate accumulation.

There is an apparent need to reduce the amounts of major metabolites, i.e. ethanol, acetate, and lactate. This could be achieved by knocking out relevant native genes in the hope to redirect the carbon flux into the 1-propanol-producing pathway. While deletions of both *adhE* and *pta* were previously found to improve succinate titers [28], these mutations abolished 1-propanol production in our study (data not shown). Deletion of *pta* resulted in the channeling of the carbon flux towards lactate accumulation. In addition, heterologous expression of *E. coli* AdhE or other ADH homologs failed to complement the *adhE* genomic knockout in terms of restoring 1-propanol production, potentially due to unknown perturbations in the metabolite pool or gene regulation. While the lactate level was significantly reduced for the *ldhA* null mutants, they produced considerable levels of both acetate and ethanol, thus reducing the carbon flux towards 1-propanol production (Table 3). Nonetheless, the *ldhA* mutation was deemed beneficial since it offers an additional NADH source and greatly reduces the acidification of the medium, thus improving cell growth.

Another critical factor limiting the production of 1-propanol (and other desired metabolites, such as succinate [28] and malate [29]) is the energetically favored diversion of carbon flux at the node of PEP towards pyruvate, resulting in the production of the major metabolites ethanol, lactate, and acetate. Blocking the production of one of these major metabolites (i.e. lactate, acetate, or ethanol) causes the accumulation of the others without improving the overall production of 1-propanol since these major metabolites all share the same precursor of pyruvate. Therefore, the implementation of a “driving force” diverting the carbon flux from pyruvate to OAA appears to be inevitable. Several metabolic engineering strategies to improve this are currently under our investigation Since a considerable amount of succinate accumulated in the extracellular medium potentially due to the poor affinity of succinate to SucCD (K_*m*_ of ~0.25 mM with succinyl-CoA as the substrate in comparison to K_*m*_ of ~4 mM with succinate as the substrate [30]), we are also identifying novel succinyl-CoA synthethases with a higher affinity for succinate to alleviate this limitation in 1-propanol production.

## Conclusions

In this study, we demonstrated the manipulation of the homologous Sbm operon for extended dissimilation of succinate in *E. coli*, leading to 1-propanol production. Using the engineered *E. coli* strains for anaerobic cultivation in a shaker, 1-propanol titers up to 150 mg/L could be obtained. However, ethanol, acetate, and lactate represented the major metabolites, potentially limiting the productivity of 1-propanol. To improve the efficiency and applicability of this biocatalytic system, further studies have to be conducted to derive superior production strains by eliminating key conversion bottlenecks, metabolic imbalances, and undesirable byproducts as well as to optimize gene expression and culture conditions.

## Methods

### Plasmid construction

All plasmids and primers used in this study are listed in Table 4. Genomic DNA from various bacterial strains was isolated using the Blood & Tissue DNA Isolation Kit (Qiagen, Hilden, Germany). Standard recombinant DNA technologies for gene cloning [31] were applied. Various DNA polymerases, restriction endonucleases, T4 DNA ligase, and Antarctic phosphatase were obtained from New England Biolabs (Ipswich, MA). All oligonucleotides were obtained from Integrated DNA Technologies (Coralville, IA). DNA sequencing was conducted in the Centre for Applied Genomics at the Hospital for Sick Children (Toronto, Canada).

**Table 4:** Hosts strains, plasmids and primers

**Name**	**Description, relevant genotype or primer sequence (5′ → 3′)**	**Reference**
***E. coli *host strains**		
HST08	F-, *endA1*, *supE44*, *thi-1*, *recA1*, *relA1*, *gyrA96*, *phoA*, *Φ80d lacZ*Δ*M15*, Δ(*lacZYA – argF*) *U169*, Δ(*mrr* – *hsdRMS* – *mcrBC*), *ΔmcrA*, λ–	Takara Bio, Shiga, Japan
MC4100	F-, [*araD139*]B/r, Del(*argF-lac*)169, λ–-, e14-, flhD5301, Δ(*fruK-yeiR*)*725*(*fruA25*), *relA1*, *rpsL150*(strR), *rbsR22*, Del(*fimB-fimE*)*632*(::IS1), *deoC1*	[32]
BW25141	F-, Δ(*araD-araB*)567, *ΔlacZ4787*(::*rrnB-3*), Δ(*phoB-phoR*)*580*, *λ*-, *galU95*, Δ*uidA3*::pir+, *recA1*, *endA9*(del-ins)::FRT, *rph-1*, Δ(*rhaD-rhaB*)568, *hsdR514*	[33]
BW25113	F-, Δ(araD-araB)567, ΔlacZ4787(::rrnB-3), λ-, rph-1, Δ(rhaD-rhaB)568, hsdR514	[33]
WT-∆*adhE*	*adhE* null mutant of BW25113	This study
WT-*∆ldhA*	*ldhA* null mutant of BW25113	This study
WT-∆*pta*	*pta* null mutant of BW25113	This study
**Plasmids**		
pCP20	FLP^+^, λ cI857^+^, λ p_R_ Rep(pSC101 ori)^ts^, Ap^R^, Cm^R^	[34]
pKD46	RepA101^ts^, Ap^R^, *araC*-P_araB_::*gam*-*bet*-*exo*	[33]
pK184	p15A ori, Km^R^, P_*lac*_::*lacZ’*	[35]
pBBR1MCS-3	broad host range ori, Tc^R^, P_*lac*_::*lacZ’*	[36]
pUC19	ColE1 ori, Ap^R^, P_*lac*_::*lacZ’*	Invitrogen, Corp., Carlsbad, CA
pK-scpAKB	From pK184, P_*lac*_:: *sbm*-*ygfD*-*ygfG*	This study
pK-scpAB	From pK184, P_*lac*_:: *sbm*-*ygfG*	This study
pB-sucCD	From pBBR1MCS-3, *araC*-P_*araB*_::*sucCD*	This study
pU-adhE(EC)	From pUC19, P_*lac*_::*adhE*(EC)	This study
pU-adhE2(CA)	From pUC19, P_*lac*_::*adhE2*(CA)	This study
pU-adhE1(CA)	From pUC19, P_*lac*_::*adhE1*(CA)	This study
pU-adhE^MUT^(EC)	From pUC19, P_*lac*_::*adhE* Glu568Lys(EC)	This study
pU-adhP(EC)	From pUC19, P_*lac*_::*adhP*(EC)	This study
pU-yqhD(EC)	From pUC19, P_*lac*_::*yqhD*(EC)	This study
**Primers**		
v-adhE	AATCTTGCTTACGCCACCTGGAAGTG; CGAACGGTCGCATGAGCAGAAAGCG	This study
v-pta	GGCATGAGCGTTGACGCAATCAACA; GATCCTGAGGTTAATCCTTCAAACG	This study
v-ldhA	TCATCAGCAGCGTCAACGGC; ATCGCTGGTCACGGGCTTACCGTT	This study
m-adhE	CATCCGGAAACTCACTTCGAAAAGCTGGCGCTG; CAGCGCCAGCTTTTCGAAGTGAGTTTCCGGA	This study
c-scpAB	CCATGATTACGAATTCGCAACAGCTTGCCAACAAGGA; TACCGAGCTCGAATTCTTAATGACCAACGAAATTAGGTTTA	This study
c-argK	GCTCTAGAATGTCTTATCAGTATGTTAAGG; GCTCTAGATTAATCATGATGCTGGC	This study
c-paraB	CCGCTCTAGATATTTAGAAAAATAAACAAATAGGGGTTCC; TTGTTTTGCCTGATATTCATGTAAGTTCATTTTTTATAACCTCCTTAGAGCTCGAATTCC	This study
c-sucCD	ATGAACTTACATGAATATCAGGCAAAACAA; CCCCCCTCGAGTTATTTCAGAACAGTTTTCAGTGCTTCACC	This study
c-adhE(EC)	CGACTCTAGAGGATCCCATGGCTGTTACTAATGTCGCTGAAC; CTCGGTACCCGGGGATCGATCGGTCAACTAATCCTTAACTGATCG	This study
c-adhE2(CA)	CGACTCTAGAGGATCCCATGAAAGTTACAAATCAAAAAGAACTAAAACAAAAGC; CTCGGTACCCGGGGATCATAGTCTATGTGCTTCATGAAGCTAATATAATGAAGCAAA	This study
c-adhE1(CA)	CGACTCTAGAGGATCCCATGAAAGTCACAACAGTAAAGGAATTAGATGAAAA; CTCGGTACCCGGGGATCTTAAGGTTGTTTTTTAAAACAATTTATATACATTTCTTTTATC	This study
c-adhP(EC)	CGACTCTAGAGGATCCCATGAAGGCTGCAGTTGTTACGAAGG; CTCGGTACCCGGGGATCTTAGTGACGGAAATCAATCACCATGC	This study
c-yqhD(CA)	CGACTCTAGAGGATCCCATGAACAACTTTAATCTGCACACCC; CTCGGTACCCGGGGATCTTAGCGGGCGGCTTCGTATATACGG	This study
c-bdhB(CA)	CGACTCTAGAGGATCCCGTGGTTGATTTCGAATATTCAATACCAACTAGAAT; CTCGGTACCCGGGGATCTTACACAGATTTTTTGAATATTTGTAGGACTTCGGA	This study

Primer notation is as follows: v- knockout primer, m- mutagenic primer and c- cloning primer. Underlined sequences within the primers denote restriction sites.

The succinyl-CoA synthetase gene (*sucCD*) from *E. coli* was cloned into the plasmid pBBR1MCS-3 for its expression under the regulation of the inducible P_*araB*_ promoter. To make this construct, *sucCD* was PCR-amplified from *E. coli* BW25141 genomic DNA using the c-sucCD primer set, whereas the *araC*-P_*araB*_ fragment was PCR-amplified from pKD46 using the c-paraB primer set. The two DNA fragments were then transcriptionally fused with splice overlap extension PCR [37] using the forward primer c-paraB and the reverse primer c-sucCD. The resulting *araC*-P_*araB*_::*sucCD* fragment was directionally cloned into the XhoI and XbaI restriction sites of pBBR1MCS-3, yielding pB-sucCD.

The fusion containing the three genes of *sbm*-*ygfD*-*ygfG* from the Sbm operon was PCR-amplified from *E. coli* BW25141 genomic DNA using the c-scpAB primer set. The amplified DNA fragment was non-directionally cloned into the EcoRI restriction site of pK184. A clone with the correct transcriptional orientation of the *sbm*-*ygfD*-*ygfG* fragment with respect to the inducible P_*lac*_ promoter was selected and verified by DNA sequencing, yielding pK-scpAKB. To test the essentialness of YgfD/ArgK, PCR was used to amplify the entire pK-scpAKB construct, with the exception of *ygfD*, using the c-argK primer set. This resulted in the addition of a flanking XbaI site downstream of *sbm* and upstream of *ygfG*. XbaI digestion and relegation of this PCR product rendered plasmid pK-scpAB.

A selection of genes encoding alcohol/aldehyde dehydrogenases from various sources were respectively cloned into pUC19 as transcriptional fusions under the control of the inducible P_*lac*_ promoter. To do this, the *adhE*, *ydhD*, and *adhP* genes were amplified from *E. coli* BW25141 genomic DNA using the c-adhE(EC), c-yqhD(EC), and c-adhP(EC) primer sets, respectively. The resulting PCR products were individually fused with the BamHI-linearized pUC19 using the In-Fusion PCR Cloning System (Clonetech Laboratories Inc., Mountainview, CA) to yield pU-adhE(EC), pU-yqhD(EC), and pU-adhP(EC), respectively. Similarly, the *adhE2*, *adhE1*, and *bdhB* genes were PCR-amplified from *Clostridium acetobutylicum* ATCC 824 genomic DNA using the c-adhE2(CA), c-adhE1(CA), and c-bdhB primer sets, respectively. The resulting PCR products were individually fused with the BamHI-linearized pUC19 to yield pU-adhE2(CA), pU-adhE1(CA), and pU-bdhB(CA), respectively. Plasmid pU-adhE^MUT^(EC) was derived from pU-adhE(EC) by generating a Glu568Lys mutation within the *adhE* coding sequence using the Phusion Site-directed Mutagenesis Kit (New England Biolabs) with the m-adhE primer set and the point-mutation was screened based on the loss of a unique SapI restriction site. Similar to a previous approach [26], pU-adhE^MUT^(EC) was used to express an aero-tolerant *E. coli* alcohol/acetaldehyde dehydrogenase mutant.

### Bacterial strains and chromosomal manipulation

A selection of *E. coli* host strains and host/vector systems used in this study are listed in Tables 4 and 5, respectively. BW25141 was used to provide wild-type (WT) genetic backgrounds for 1-propanol production. HST08 was used for molecular cloning. Various host gene deletions (e.g. *adhE*, *pta*, and *ldhA*) were introduced to BW25141 by P1-phage transduction [31] using proper Keio Collection strains (CGSC, Yale University) as donors [38]. The co-transduced Km^R^-FRT gene cassette was removed using pCP20 [33]. *E. coli* strain MC4100 was used as a control strain for all P1 phage transductions. The genotypes of derived knockout strains were confirmed with colony PCR using appropriate primer sets (e.g. v-adhE, v-pta, and v-ldhA).

**Table 5:** ***E. coli *strains containing variants of the synthetic 1-propanol pathway used in this study**

**Strain**	***E. coli *host**	**Plasmid 1**	**Plasmid 2**	**Plasmid 3**
WT2	BW25141	pK-scpAKB	pB-sucCD	—
WT-adhE2(CA)^2^	BW25141	pK-scpAKB	—	pU-adhE2(CA)
WT-adhE2(CA)^3^	BW25141	pK-scpAKB	pB-sucCD	pU-adhE2(CA)
WT-adhE2(CA)^3^-∆ygfD	BW25141	pK-scpAB	pB-sucCD	pU-adhE2(CA)
WT-adhE1(CA)	BW25141	pK-scpAKB	pB-sucCD	pU-adhE1(CA)
WT-adhE^MUT^(EC)	BW25141	pK-scpAKB	pB-sucCD	pU-adhE^MUT^(EC)
WT-adhP(EC)	BW25141	pK-scpAKB	pB-sucCD	pU-adhP(EC)
WT-yqhD(EC)	BW25141	pK-scpAKB	pB-sucCD	pU-yqhD(EC)
WT-bdhB(CA)	BW25141	pK-scpAKB	pB-sucCD	pU-bdhB(CA)
∆*ldhA*-adhE(EC)	WT-∆*ldhA*	pK-scpAKB	pB-sucCD	pU-adhE(EC)
∆*ldhA*-adhE2(CA)	WT-∆*ldhA*	pK-scpAKB	pB-sucCD	pU-adhE2(CA)
∆*ldhA*-adhE1(CA)	WT-∆*ldhA*	pK-scpAKB	pB-sucCD	pU-adhE1(CA)
∆*ldhA*-adhE^MUT^ (EC)	WT-∆*ldhA*	pK-scpAKB	pB-sucCD	pU-adhE^MUT^(EC)
∆*ldhA*-adhP(EC)	WT-∆*ldhA*	pK-scpAKB	pB-sucCD	pU-adhP(EC)
∆*ldhA*-yqhD(EC)	WT-∆*ldhA*	pK-scpAKB	pB-sucCD	pU-yqhD(EC)
∆*ldhA*-bdhB(CA)	WT-∆*ldhA*	pK-scpAKB	pB-sucCD	pU-bdhB(CA)

### Media and cultivation

All chemicals for medium components were obtained from Sigma-Aldrich Co. (St Louis, MO) except yeast extract and tryptone, which were obtained from BD Diagnostic Systems (Franklin Lakes, NJ). When required, antibiotics at a proper concentration were used: 100 μg/mL carbenicillin, 50 μg/mL kanamycin, and 20 μg/mL tetracycline. For multi-plasmid systems, the concentration of each antibiotic was reduced to half to avoid negative impacts on growth. Isopropyl-beta- d-thiogalactopyranoside (IPTG) (1 mM) and l-arabinose (10 mM) were used to induce gene expression respectively regulated by the P_*lac*_ and P_*araB*_ promoters.

For all cultivation experiments, *E. coli* strains (stored as glycerol stocks at -80°C) were streaked on LB plates with appropriate antibiotics and incubated for 16 h at 37°C. Single colonies were picked from LB plates to inoculate 25-mL LB media with appropriate antibiotics in 125-mL conical flasks. The cultures were grown in a rotary shaker at 250 rpm and 37°C to reach an optical cell density at 600 nm (OD_600_) of 0.7. Four milliliter of the seed culture was used to inoculate 400-mL LB media with appropriate antibiotics in 1-L conical flasks. This second seed culture was also shaken at 250 rpm and 37°C to reach an OD_600_ of 0.7. Cells were collected by centrifugation at 6,000 × g and 4°C for 20 min and the cell pellets were transferred into a controlled anaerobic atmosphere (85% N_2_, 10% H_2_, and 5% CO_2_) in an anaerobic chamber (Plas-Labs Inc., Lansing, MI). Cell pellets were washed and resuspended in reduced modified M9 minimal media [6 g/L Na_2_HPO_4_, 3 g/L KH_2_PO_4_, 0.5 g/L NaCl, 1g/L NH_4_Cl, 1 mM MgSO_4_, 0.1 mM CaCl_2_, 10 mM NaHCO_3_, 10 mg/L vitamin B_1_, and 0.2 μM cyanocobalamin (vitamin B_12_)] containing appropriate carbon sources, 5 g/L yeast extract, appropriate antibiotics and inducers, and 1000X trace metal mix A5 (2.86 g/L H_3_BO_3_, 1.81 g/L MnCl_2_•4H_2_O, 0.222 g/L ZnSO_4_•7H_2_O, 0.39 g/L Na_2_MoO_4_•2H_2_O, 0.079 g/L CuSO_4_•5H_2_O, 49.4 mg/L Co(NO_3_)_2_•6H_2_O). Cells were resuspended to a final OD_600_ of 15 unless specified otherwise. While most oxygen in the modified M9 minimal media was purged by autoclaving, trace oxygen was reduced using a palladium catalyst attached to the heating unit of the anaerobic chamber. The anaerobic condition of the medium was monitored using resazurin, which was added at 1 mg/L. Suspended cultures were then transferred into 50-mL screw-capped conical flasks and sealed with Parafilm, before being removed from the anaerobic chamber and placed in a rotary shaker running at 250 rpm 37°C. Cultures were unsealed and analyzed after 3 d.

### Analytical procedures

Culture samples were appropriately diluted with an isotonic saline solution for measuring the optical cell density (OD_600_) using a spectrophotometer (DU520, Beckman Coulter, Fullerton, CA). For HPLC and NMR analyses, culture samples were centrifuged for 3 min at 13,000 × g to recover the supernatant fraction which was filtered with a 0.2 μM syringe filter prior to being stored at -20°C.

### HPLC analysis

Extracellular metabolites were analyzed using HPLC (LC-10ATVP, Shimadzu, Kyoto, Japan) equipped with an Aminex HPX87 column (BioRad Laboratories, Hercules, CA) and a refractive index detector (RID-10A, Shimadzu, Kyoto, Japan). The column temperature was maintained at 65°C when conducting analysis. The mobile phase was 5 mM H_2_SO_4_ (pH 2.0) running at 0.6 mL/min. The RID was connected to an integrator (C-R8A, Shimadzu, Kyoto, Japan) for chromatographic data processing. Pure samples of various metabolites with concentrations ranging from 0.02 to 12.0 g/L were used as standards for calibration. Cell-free fermentation samples were subjected to filtration treatment prior to their injection for HPLC analysis.

### NMR analysis

#### NMR sample preparation

Extracellular medium samples were diluted in 10% v/v with an internal standard composed of 99.9% D_2_O with 5 mM 2,2-Dimethyl-2-silapentane-5-sulfonate (DSS) serving as a chemical shape indicator (CSI) and 0.2% w/v sodium azide (NaN_3_) to inhibit bacterial growth. The diluted samples were subsequently transferred to 5-mm NMR tubes (NE-UL5-7, New Era Enterprises Inc., Vineland, NJ). Spectra were acquired by a 1D NOESY pulse sequence on a Bruker Avance 600.13 MHz spectrometer with a TXI 600 Probe (Bruker Canada Ltd., Toronto, Canada).

#### Spectra processing and compound identification

Following acquisition, spectra were imported into Chenomx NMR Suite 7.5 (Chenomx Inc., Edmonton, Alberta, Canada) for data processing with phase, baseline, shim, and shape corrections being carried out. An average sample pH of 5.2 measured during fermentation was applied as a reference for metabolite identification. Following spectral processing, various extracellular metabolites were identified by targeted profiling. Since the compound database associated with Chenomx NMR Suite 7.5 software did not include 1-propanol (Figure 5) or propionaldehyde, the 'compound builder’ application was used to implement the hydrogen spectra and unique peaks of these compounds.

## Abbreviations

Δ: Deletion; []: Denotes plasmid-carrier state; accAB: Genes encoding acetyl acetyl-CoA carboxylase; ackA: Acetate kinase; ADH(s): Alcohol dehydrogenase(s); adhE: Gene encoding acetaldehyde dehydrogenase/alcohol dehydrogenase; adhE1: Gene encoding bifunctional acetaldehyde-CoA/alcohol dehydrogenase; adhE2: Gene encoding bifunctional acetaldehyde-CoA/alcohol dehydrogenase; adhP: Gene encoding alcohol/acetaldehyde dehydrogenase, 1-propanol preferring; aldBA: Genes encoding aldehyde dehydrogenase; Ap: Ampicillin; argK/ygfD: Gene encoding arginine kinase; bdhB: Gene encoding butanol dehydrogenase; bla: Gene encoding Ap^R^ gene; cI857: Gene encoding temperature-sensitive λ repressor; DO: Dissolved oxygen; fbaB: Genes encoding fructose-bisphosphate aldolase; flp: Gene encoding *Saccharomyces cerevisiae* Flp recombinase; frdABCD: Fumarate reductase; FRT: Flp recombination target; fumABC: Genes encoding fumarate hydratase; gapA: Gene encoding glyceraldehyde-3-phosphate dehydrogenase; glk: Gene encoding glucokinase; gloAB: Gene encoding glyoxalase I; glpK: Gene encoding glycerol kinase; gltA: Gene encoding citrate synthase; gpmA: Gene encoding phosphoglyceromutase I; gpsA: Gene encoding glycerol-3-phosphate dehydrogenase; HPLC: High-performance liquid chromatography; icd: Gene encoding isocitrate dehydrogenase; IPTG: Isopropyl β- d-thiogalactopyranoside; kan: Gene encoding Km^R^; Km: Kanamycin; KmR FRT: Cassette carrying Km^R^ marker, LB, lysogeny broth; ldhA: Gene encoding lactate dehydrogenase; mdh: Gene encoding malate dehydrogenase; mgr: Gene encoding glyceraldehyde 3-phosphate reductase; mgsA: Gene encoding methylglyoxal synthase; NMR: Nuclear magnetic resonance; OAA: Oxaloacetate; PEP: Phosphoenolpyruvate; pepC: Phosphoenolpyruvate carboxylase; pfkAB: Gene encoding 6-phosphofructokinase; pflB: Gene encoding pyruvate formate lyase I; pgi: Gene encoding glucosephosphate isomerase; pgk: Gene encoding phosphoglycerate kinase; poxB: Gene encoding pyruvate dehydrogenase; prpC: Gene encoding 2-methylcitrate synthase, *pta*, gene encoding phosphate acetyltransferase; ptc: Gene encoding phosphoenolpyruvate carboxylase; ptsG: Gene encoding glucose-specific phosphotransferase permease; pyc: Gene encoding pyruvate carboxylase; pykFA: Gene encoding pyruvate kinase; R: Resistant/resistance; S: Sensitive/sensitivity; sbm/scpA: Methylmalonyl-CoA mutase; sdhAB: Gene encoding succinate dehydrogenase; sucCD: Gene encoding succinyl-CoA synthetase; Tc: Tetracycline; TCA: Tricarboxylic acid cycle; tpiA: Gene encoding triosephosphate isomerase; tetAR: Genes encoding Tc^R^ and Tc repressor; tktAB: Genes encoding transketolase; ts: Temperature sensitive; WT: Wild type; ygfG/scpB: Gene encoding methylmalonyl-CoA decarboxylase; ygfH/scpC: Gene encoding propionyl-CoA:succinate-CoA transferase; yqhD: Gene encoding aldehyde reductase.

## Competing interests

The authors declare that they have no competing interests.

## Authors’ contributions

KS conceived the study, designed and carried out the experiments, and drafted the manuscript. LA participated in the experimental design, performed data interpretation and analysis, and helped to draft the manuscript. XL and AW participated in the experimental design. EJB and MGA carried out NMR spectroscopic analysis. MMY and CPC conceived, supervised, and managed the study, as well as helped to draft the manuscript. All authors read and approved the final manuscript.
